# Idiopathic spontaneous perforation of the common bile duct in an adult

**DOI:** 10.1093/jscr/rjaf794

**Published:** 2025-10-09

**Authors:** G Balamurugan, Pooja Syamala Nair, Jignesh Jatania, Kamil Wynne, Daya Singh

**Affiliations:** Department of General Surgery, South Tyneside and Sunderland NHS Foundation Trust, Harton Lane, South Shields, Tyne and Wear, NE34 0PL, United Kingdom; Department of General Surgery, South Tyneside and Sunderland NHS Foundation Trust, Harton Lane, South Shields, Tyne and Wear, NE34 0PL, United Kingdom; Department of General Surgery, South Tyneside and Sunderland NHS Foundation Trust, Harton Lane, South Shields, Tyne and Wear, NE34 0PL, United Kingdom; Department of General Surgery, South Tyneside and Sunderland NHS Foundation Trust, Harton Lane, South Shields, Tyne and Wear, NE34 0PL, United Kingdom; Department of General Surgery, South Tyneside and Sunderland NHS Foundation Trust, Harton Lane, South Shields, Tyne and Wear, NE34 0PL, United Kingdom

**Keywords:** spontaneous common bile duct perforation, idiopathic biliary perforation, biliary peritonitis, endoscopic biliary stenting, diagnostic laparoscopy

## Abstract

Spontaneous perforation of the common bile duct (SPCBD) is an extremely rare clinical entity in adults, typically idiopathic in origin, though potential causes include distal obstruction, reflux of pancreatic secretions, intramural vascular thrombosis, infection, or direct erosion by stones. We present a rare case of idiopathic SPCBD in an adult who had undergone a previous cholecystectomy. Imaging findings revealed biliary dilatation and a fluid collection extending to the paracolic gutter, posing a challenge in determining whether it was a collection related to pancreatitis or other cause. Laparoscopy for peritonitis revealed a biliary collection, which was successfully drained. Following this, an Endoscopic Retrograde Cholangio-Pancreatography identified a bile leak from the common bile duct, leading to a diagnosis of idiopathic SPCBD. The patient was successfully managed with biliary stenting. This case emphasizes the critical role of multidisciplinary team discussions and the value of collaborative approaches in treating such complex conditions.

## Introduction

Spontaneous perforation of the common bile duct (SPCBD) is a rare entity, predominantly observed in infants because of congenital abnormalities affecting the biliary tract [1]. Its manifestation in the adult population is exceedingly rare [2]. The predominant cause for SPCBD in adults is idiopathic [3]. Other potential etiological factors contributing to SPCBD include elevated intraluminal pressure due to conditions such as stones, strictures, tumors, or parasites causing distal obstruction, as well as reflux of pancreatic secretions, thrombosis of intramural vessels leading to necrosis, intramural infection, congenital weakness of the biliary wall, or direct erosion by stones [4]. In this report, we present a case of idiopathic SPCBD in an adult that was managed successfully.

## Case report

A female patient in her fifth decade presented to the Emergency Department with a two-day history of right upper abdominal pain radiating to her back, along with vomiting and fever. She had a past surgical history of cholecystectomy, along with an appendectomy performed three decades ago. She had a history of Bipolar II disorder, managed with quetiapine and venlafaxine. Social history revealed daily consumption of a bottle of wine and 20 cigarettes. There were no allergies. Before her admission, she had no recurrent abdominal symptoms or pain.

**Figure 1:**
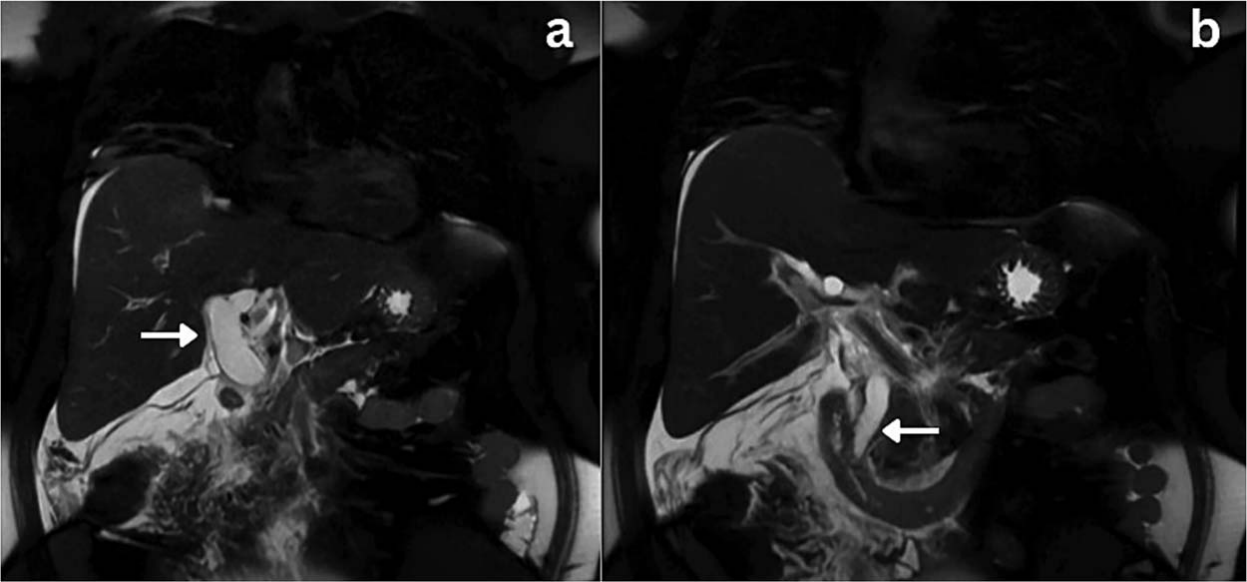
Coronal section MRCP imaging; a) Gross biliary dilatation (arrow), b) smooth tapering of the bile duct with no evidence of stones (arrow).

**Figure 2:**
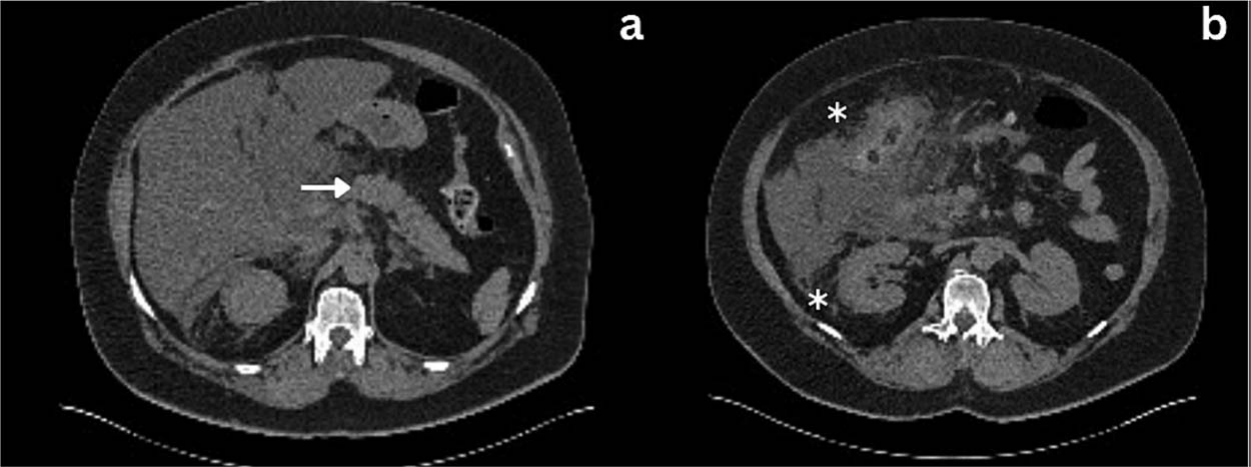
Axial section CT imaging; a) Normal pancreas with no evidence of inflammation or malignancy (arrow), b) extrahepatic biliary collection (asterisks).

She was admitted, resuscitated, and started on intravenous antibiotics. Considering her previous history of cholecystectomy and deranged Liver Function Tests (LFTs), a clinical diagnosis of cholangitis was made. Magnetic retrograde cholangio-pancreatography (MRCP) was done, which showed significant dilatation of the biliary system extending down to the ampulla with the common hepatic duct (CHD) diameter of 23 mm with a normal pancreatic duct. Additionally, there was florid upper abdominal edema and fluid tracking along the right paracolic gutter (Fig. 1). There was no evidence of any common bile duct (CBD) stone or lesion to explain this dilatation. To further explore the underlying cause, a computed tomography (CT) pancreas was performed, which revealed no signs of pancreatitis nor any distal CBD or pancreatic lesions. The presence of fluid in the subhepatic and right paracolic regions likely indicated a bile leak (Fig. 2), but fluid related to pancreatitis could not be excluded. The findings were deliberated upon with the specialist hepatopancreaticobiliary (HPB) team and Specialist gastrointestinal (GI) radiologists. Despite extensive discussion and review with the radiologist, the nature of the fluid was not identified through the two imaging modalities employed. There was no obvious pancreatitis. A decision was reached to continue conservative management and opt for operative intervention in the event of clinical deterioration.

**Figure 3:**
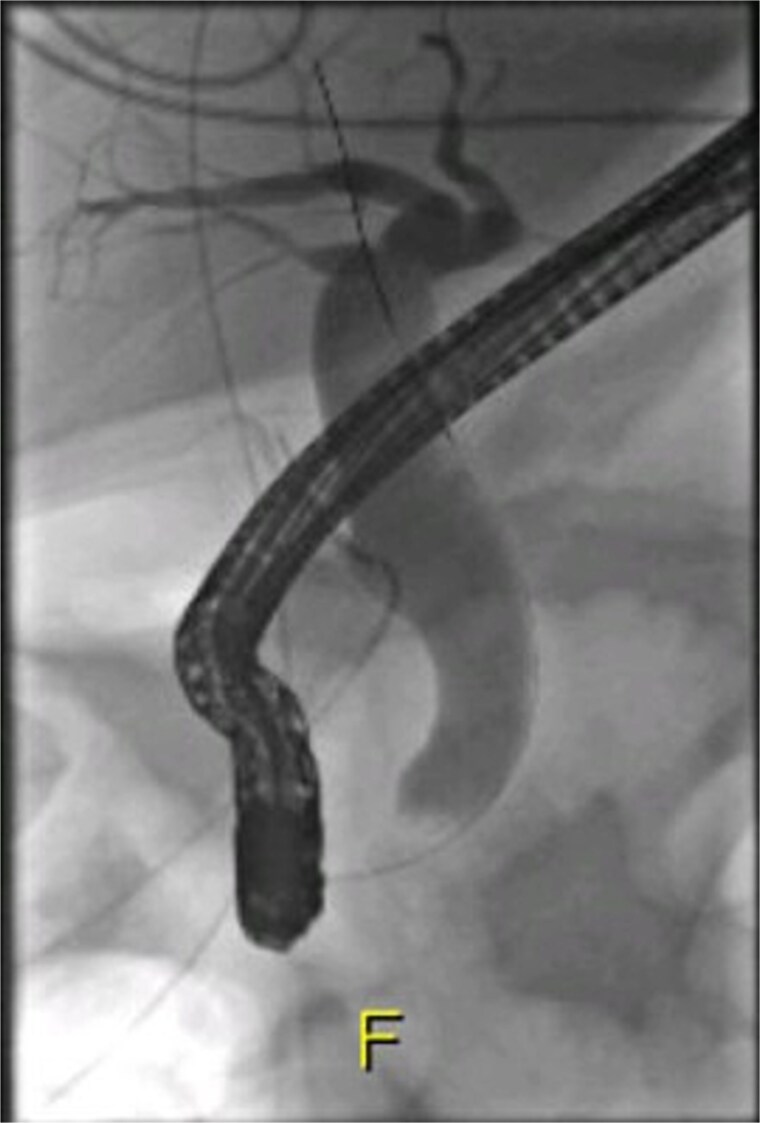
ERCP imaging reveals contrast leak in the cystic duct stump region.

**Figure 4:**
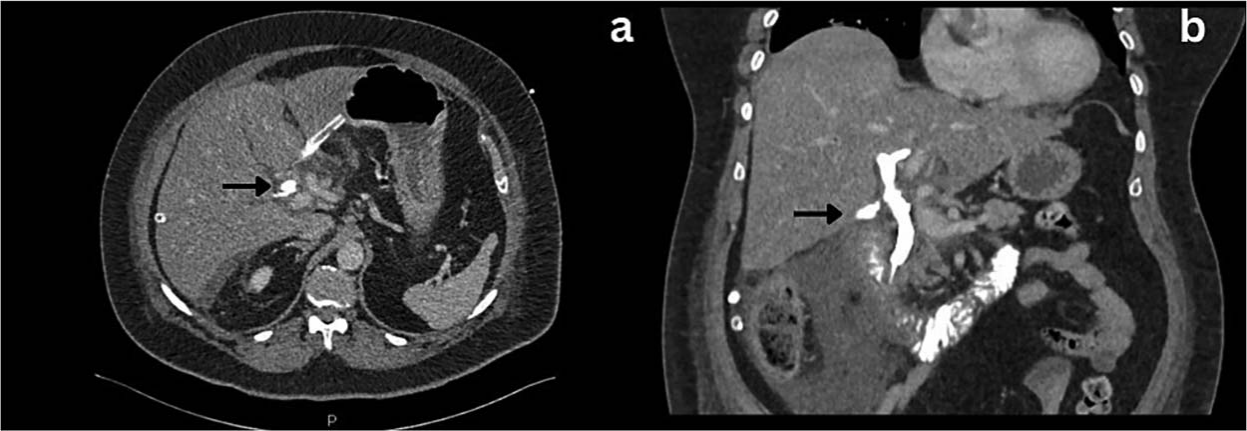
Axial and coronal section CT imaging revealing contrast leak from the bile duct; a) Contrast leak from the posterior aspect of CBD (arrow). b) Contrast extravasation into the subhepatic drain (arrow).

Following a brief clinical response to the initial conservative management for 24 hours, the patient started deteriorating and developed generalized peritonitis with worsening of inflammatory markers (C-reactive protein escalated to 438 mg/l). Consequently, she underwent an emergency diagnostic laparoscopy as the cause of peritonitis was not clear. Intraoperatively, it was observed that there was free bile in the peritoneal cavity, more so around the liver and the right paracolic gutter. There was also a bile collection behind the right colon and hepatic flexure of the colon, raising the possibility of a retroperitoneal source. The hepatic flexure of the colon was mobilized to assess the source of the leak. No obvious ongoing leak was noticed. As a precautionary measure, an Upper GI endoscopy was conducted intra-operatively to rule out any gastroduodenal perforation, as no obvious source of bile leak was seen from the biliary tree. This revealed the presence of bile in the stomach and confirmed normal anatomy up to the third part of the duodenum. The remaining intra-abdominal organs appeared unremarkable. Thorough peritoneal lavage was given, and 22 Fr abdominal drains were placed in the subhepatic, para-hepatic space, and pelvis, respectively, as the source of leak was not found. The following day, an endoscopic retrograde cholangiopancreatography (ERCP) was performed, which identified a small leak around the area of the cystic duct stump (Fig. 3). In response, a 5 cm-sized 7 Fr double pigtail stent was inserted into the CBD. A post-ERCP CT scan was performed to evaluate the contrast leak. The CT imaging revealed an extraluminal contrast leak originating from a defect in the posterior CBD (Fig. 4). Notably, the cystic duct stump remained intact and anterior, establishing the diagnosis of Spontaneous biliary leak from the CBD.

**Figure 5:**
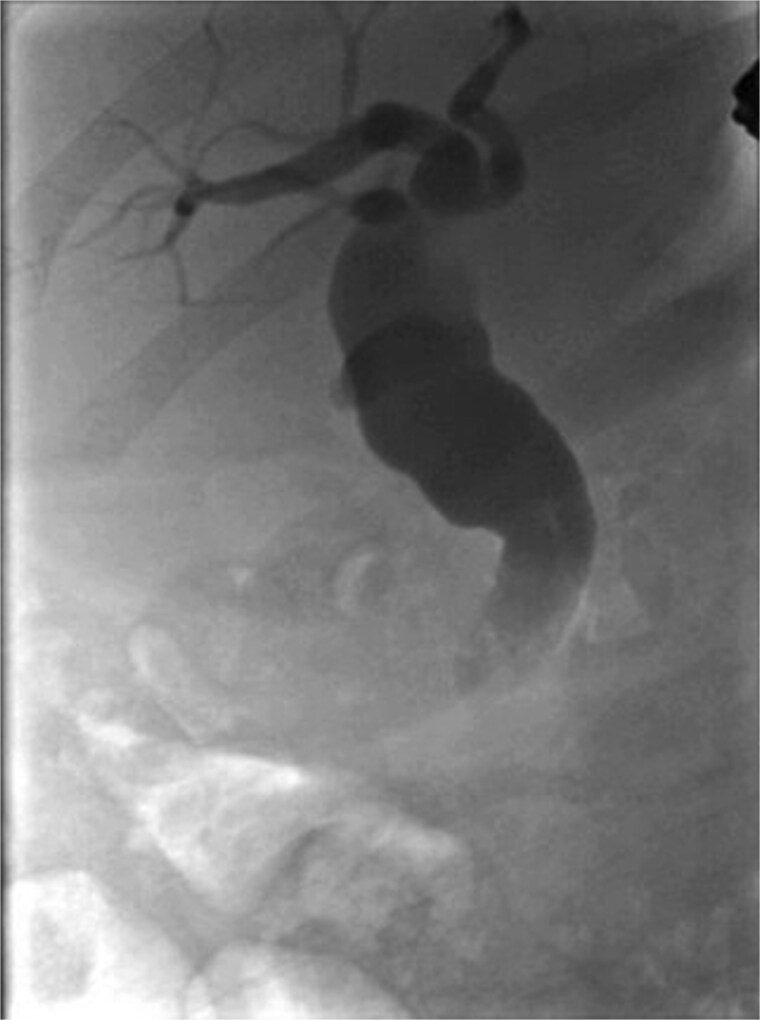
ERCP imaging after stent removal depicting no contrast leak.

Following surgical intervention, the patient was admitted to the intensive care unit (ICU), where she was closely monitored for three days before being stepped down to the ward. During this time, her labs normalized, and drain output decreased steadily. The drain was removed on post-operative day (POD) 11, and she was subsequently discharged. Notably, the cytology of CBD brushings did not reveal any malignant cells. A repeat ERCP was performed after 8 weeks, which revealed no contrast leak, indicating spontaneous healing (Fig. 5), and the biliary stent was removed. Successive clinic follow-up at 6 months post-treatment indicated that the patient remained completely asymptomatic and exhibited excellent clinical recovery.

## Discussion

The first documented biliary rupture was reported by John Freeland in 1882, describing hepatic duct rupture in a 65-year-old woman due to diverticula and stones [5]. Since then, numerous reports have contributed to our understanding of diagnosing and managing this rare condition. The presentation of patients with SPCBD can vary widely, with ~20% presenting acutely and 80% insidiously. Acute cases often present with progressive jaundice and painless abdominal distension, while insidious cases mimic an acute abdomen, manifesting as generalized abdominal pain, distension due to bilious ascites, vomiting, fever, jaundice, elevated bilirubin levels, or even shock. Additionally, patients may develop a perihepatic collection or abscess if bile is localized [4].

Diagnosing SPCBD is challenging due to its resemblance to other biliary pathologies. Bouzid *et al.*'s review outlined various investigative methods, including traditional ultrasound (US), CT, MRCP, scintigraphy, and paracentesis [3]. In our case, diagnostic laparoscopy was performed due to clinical deterioration to exclude other serious pathologies. A high index of suspicion is crucial when conventional imaging is inconclusive.

The management of SPCBD encompasses a range of approaches, from conservative measures to minimally invasive interventions and aggressive surgical drainage. A multidisciplinary team approach, as exemplified in our case, facilitates the development of an appropriate treatment plan based on the patient's specific clinical condition. The feasibility of primary closure depends on the size of the perforation, as well as the presence of significant inflammatory reactions or distal obstruction. In selected cases where these factors are minimal, primary closure may be considered. Alternatively, for very small perforations, ERCP and stenting can serve as an alternative option. By providing decompression, this approach facilitates the automatic healing of the perforation. However, if the perforation is extensive or not amenable to primary closure, it may require T-tube drainage or bilio-enteric anastomosis [6].
